# Enteric Fever Diagnosis: Current Challenges and Future Directions

**DOI:** 10.3390/pathogens10040410

**Published:** 2021-04-01

**Authors:** Durga P. Neupane, Hari P. Dulal, Jeongmin Song

**Affiliations:** Department of Microbiology and Immunology, Cornell University, Ithaca, NY 14853, USA; dn332@cornell.edu (D.P.N.); hpd32@cornell.edu (H.P.D.)

**Keywords:** enteric fever diagnosis, typhoid fever diagnosis, *Salmonella*, *Salmonella* Typhi, enteric fever surveillance, antimicrobial resistance

## Abstract

Enteric fever is a life-threatening systemic febrile disease caused by *Salmonella enterica* serovars Typhi and Paratyphi (*S.* Typhi and *S.* Paratyphi). Unfortunately, the burden of the disease remains high primarily due to the global spread of various drug-resistant *Salmonella* strains despite continuous advancement in the field. An accurate diagnosis is critical for effective control of the disease. However, enteric fever diagnosis based on clinical presentations is challenging due to overlapping symptoms with other febrile illnesses that are also prevalent in endemic areas. Current laboratory tests display suboptimal sensitivity and specificity, and no diagnostic methods are available for identifying asymptomatic carriers. Several research programs have employed systemic approaches to identify more specific biomarkers for early detection and asymptomatic carrier detection. This review discusses the pros and cons of currently available diagnostic tests for enteric fever, the advancement of research toward improved diagnostic tests, and the challenges of discovering new ideal biomarkers and tests.

## 1. Introduction

Enteric fever, referring to typhoid fever and paratyphoid fever, is a common bacterial disease with high morbidity and mortality rates in low- to middle-income countries in Asia, Africa, and South America, associated with limited proper sanitation and safe drinking water supply [1,2]. The World Health Organization (WHO) estimates up to 21 million enteric fever cases and 161,000 deaths each year worldwide. However, the actual burden of the disease is unknown since this estimate was extrapolated from the limited number of surveillance studies using current diagnostic measures [3]. Among over 2600 closely-related *Salmonella enterica* serovars, human-restricted *Salmonella enterica* serovars Typhi and Paratyphi A, B, and C (*S.* Typhi and *S.* Paratyphi A, B, and C) are the cause of enteric fever. Different *Salmonella* serovars, including *S.* Typhi and *S.* Paratyphi, are characterized by a distinct set of their surface antigens: lipopolysaccharide O (somatic), flagellar H, and virulence-capsule (Vi) antigens [4]. Based on their host-specificity and disease outcomes, *S. enterica* are grouped into typhoidal and nontyphoidal *Salmonella* serovars (NTS). The majority of NTS serovars represented by *S.* Typhimurium and *S.* Enteritidis can infect humans and animals and cause a self-limiting gastrointestinal Salmonellosis in humans, with some exceptions of NTS causing invasive disease [5,6,7].

In addition to diagnostic challenges associated with closely related *Salmonella* serovars, the infection route and some clinical presentations are also shared among *Salmonella* serovars. *Salmonella* serovars are transmitted through the fecal–oral route after the ingestion of contaminated food and water. The incubation period of enteric fever is approximately 8–14 days [8], while the duration and severity of the disease are affected by the types of bacterial strains and doses as well as host immune responses [9,10,11]. Typhoid and paratyphoid fevers are clinically indistinguishable from each other. They can present with comparable severity of the complications, although typhoid fever is more prevalent than paratyphoid fever in most endemic areas [12,13]. For instance, clinical presentations such as high fever, headache, malaise, anorexia, rapid pulse, leukopenia, thrombocytopenia, abdominal discomfort, and neurological complications are not specific to enteric fever [14,15], making a clinical-presentation-based diagnosis difficult. Viral (e.g., dengue, influenza), parasitic (e.g., malaria, typhus, leishmaniosis), and other bacterial (e.g., brucellosis, tuberculosis) infections that are also common in endemic areas may develop similar symptoms [16]. The current diagnostic tests cannot reliably distinguish enteric fever from others.

The global spread of multidrug-resistant (MDR) *Salmonella* and the emergence of extensively drug-resistant (XDR) *Salmonella* also support the need for improved diagnostic tests, as well as new treatment strategies that are alternatives to current antibiotics. Antibiotics are primary treatment options for enteric fever, but *Salmonella* is continuously evolving to acquire plasmid, prophage, transposon, or chromosomal gene mutations to attain antibiotic-resistance. A myriad of reports has indicated the global spread of *S.* Typhi and *S.* Paratyphi strains that are resistant to all of the first-line antibiotics, ampicillin, chloramphenicol, and co-trimoxazole, collectively known as multidrug-resistance (MDR) *Salmonella* [17,18,19]. All of the identified MDR *S.* Typhi and *S.* Paratyphi carry the IncHI1 plasmid, while other antibiotic-resistant related genes found in MDR *Salmonella* can vary [20]. Haplotype-58 (H58) is the most dominant MDR *S.* Typhi strain identified in various parts of Asia and Africa and travel-related MDR cases in other countries [21,22,23,24,25].

The emergence and spread of *Salmonella* strains resistant to the second line of drugs have also been reported [25,26]. Resistance to fluoroquinolones has been acquired by chromosomal mutations in the quinolone resistance gene *qnrS* and/or quinolone resistance determining region (QRDR) harboring *gyrA, gyrB, parC,* and *parE* genes [25,26]. Resistance to third-generation cephalosporins is associated with the acquisition of several extended-spectrum β-lactamase (ESBL) genes [27]. The XDR H58 *S.* Typhi strain, resistant to ampicillin, chloramphenicol, co-trimoxazole, fluoroquinolones, and third-generation cephalosporins, was first identified in Pakistan, affecting over 300 cases in 2016 [27]. Since then, XDR *S.* Typhi infection remains prevalent in the region, and travel-related XDR *S.* Typhi infections have been reported in many other countries [28,29,30,31], indicating the rapid global spread of XDR *S.* Typhi. XDR H58 isolates harbored the IncY plasmid, carrying an ESBL-resistance gene. Azithromycin and carbapenems are “last resort” antibiotics for treating *Salmonella* infection, but the emergence of azithromycin-resistant *S.* Typhi strains and carbapenem-resistant invasive NTS has also been reported [32,33,34,35]. These observations support the urgent need of improved diagnostic, prevention, and treatment strategies to better control drug-resistant *S.* Typhi and *S.* Paratyphi.

Ideal diagnostic tests should also detect asymptomatic carriers and distinguish the infection from others. A significant population (2–5%) of recovered patients become asymptomatic chronic carriers who can shed the bacteria intermittently in their feces for years [36,37]. Chronic carriers serve as a primary reservoir of *S.* Typhi and *S.* Paratyphi that persist mainly in the gallbladder for local and global spread [37,38,39], as they are human-restricted pathogens with no other known reservoirs.

The arrival of new diagnostic tests allowing the early detection of *S.* Typhi and *S.* Paratyphi infection and the detection of chronic carriers would help eradicate enteric fever. This review discusses the pros and cons of the currently available diagnostic tests for enteric fever, notable bacterial virulence factors in the context of their potential to be used as new diagnostic biomarkers, other bacterial determinants identified by systemic approaches as promising biomarkers, and the remaining challenges of discovering new ideal biomarkers and tests.

## 2. Current Enteric Fever Diagnostics

Laboratory diagnosis is required to confirm enteric fever. Although enteric fever has been well established for more than a century, there has not been a single “ideal” laboratory diagnostic biomarker available (Table 1).

### 2.1. Bacterial-Culture-Based Diagnosis

The definitive diagnosis of enteric fever requires the isolation of bacteria from blood or bone marrow, accompanied by fever ≥38 °C for at least three days [40]. Culture remains the mainstay of diagnosis, and bacterial isolation allows us to characterize the pathogen for antibiotic resistance genes and the causation of the outbreak of disease in the particular location. Although the method has 100% specificity, it lacks sensitivity. On average, blood and bone marrow cultures have a sensitivity of ~50% and ~80%, respectively, which directly correlates with the number of viable bacteria in blood (≤1 CFU/mL) and bone marrow (~10 CFU/mL) [41,42]. Various strategies have been employed to increase the sensitivity of bacterial-culture-mediated diagnosis (Table 1). For example, supplementation of ox bile or bile salt (sodium taurocholate) to the culture media has resulted in increased bacterial isolation frequency in a shorter time [43]. More specifically, bile contents suppress the bactericidal activity of blood and lyse blood cells to release bacteria.

Similarly, sodium polyethanol sulfonate can also reduce the bactericidal activity of blood [44] and shorten the testing time required for bacteria isolation without changing the overall isolation frequency [45]. After removing serum due to its bactericidal activity, blood clot culture also exhibits increased sensitivity and rapid bacterial growth [46,47,48]. More blood volumes and additional dilutions of the specimens with media have also been used to improve bacterial detection frequency and address the sensitivity issue associated with culture-based diagnosis methods [49].

These studies indicate that the optimum ratio of blood to bacterial culture media (e.g., tryptic soy broth (TSB)) should be 1:10 or greater. The standard method involves an incubation at 37 °C and an inspection for bacterial growth for at least a week. In general, positive cultures are subcultured at 37 °C for 24 h on both nonselective enriched media (e.g., blood agar) that supports the growth of most bacteria and selective differential media (e.g., MacConkey agar, xylose lysine deoxycholate (XLD) agar) that allows the growth of bile-tolerant bacteria such as *S.* Typhi and *S.* Paratyphi for diagnosis. The use of nonselective media such as TSB or blood agar helps isolate bacterial pathogens in blood, which should be sterile in healthy individuals. The use of selective media such as MacConkey and XLD agars helps differentiate non-lactose-fermenting bacteria such as *S.* Typhi and *S.* Paratyphi from lactose-fermenting bacteria such as *E. coli* and bile-tolerant *S.* Typhi and *S.* Paratyphi from other pathogens such as Gram-positive bacteria and *E. coli*, respectively. Biochemical identification and agglutination with specific antisera tests are followed to diagnose infection with *S.* Typhi and/or *S.* Paratyphi. The basis of serotyping is described in the latter part of this paper (Section 2.3).

There are some additional challenges associated with blood-culture-based diagnostic methods. In brief, compared to the use of 5–10 mL blood samples for school-age children and adults during the first two weeks of the infection, at which the bacterial load is higher [41,50], a smaller blood volume (2–4 mL) is used for preschool children [40], which is likely associated with underdiagnosis among younger populations [51]. Prior antibiotic therapy, which remains very common in endemic areas, also hinders culture-based diagnosis [52,53]. This challenge is overcome by using bone marrow samples rather than blood for bacterial culture since bacteria in the bone marrow are unlikely cleared by antibiotic treatment [52,54]. For this reason, bone marrow culture is generally considered the gold standard for enteric fever diagnosis in endemic areas. However, this method involves an invasive procedure for sample collection and requires specialized skills and equipment to conduct.

Besides bacterial isolation from blood and bone marrow samples, in some cases, other biological samples such as rose spot, duodenal bile, stool, and urine are used for *Salmonella* isolations via culture. A rose spot culture gives ~60% sensitivity, which is a noninvasive procedure, but the occurrence of these spots is relatively rare among enteric fever patients (1–30%) [42]. Duodenal aspirate culture can provide a better diagnostic value than stool culture, but the test’s tolerance, particularly among children, hampers its use [55]. The positive results from these other biological samples are only suggestive of active disease due to chronic carriage prevalence in endemic areas. Therefore, a positive result should be interpreted in combination with other assays.

As described above, a bacterial-culture-based diagnosis is the gold-standard for enteric fever diagnosis, also allowing for antibiotic-susceptibility testing that is essential for determining a proper antibiotic treatment strategy. The primary challenges of this method include a slower turnaround time (≥48 h) required for bacterial growth and identification and the need for appropriate laboratory infrastructure, which is not necessarily common in endemic areas.

### 2.2. Bacterial Nucleic Acid Detection-Based Diagnosis

Nucleic acid detection involves polymerase chain reaction (PCR) that amplifies *Salmonella* serovar-specific DNA for diagnosis. The primary advantage of this method is the rapid turnaround time. PCR methods are advantageous because they can detect *Salmonella*-specific DNA extracted from live or dead bacteria or both. Dead bacteria in blood can result from antibiotic treatments, which is also common in some endemic areas and/or outcomes of host immune responses. The disadvantage of PCR methods includes the need for trained personnel and special equipment to conduct the PCR.

This method involves DNA extraction from patient samples followed by amplification of *Salmonella*-specific DNA sequences. The most commonly used target genes for enteric fever diagnosis include flagellin (*fliC*), Vi polysaccharide (*viaB*), 16s rRNA, heat-shock protein (*groEL*), cytotoxin (*clyA*), and other conserved genes. Due to the lack of a standard reference method, the accuracy of the test is generally calculated based on blood culture results. Various studies have demonstrated that the sensitivity ranges from 40–100%, as shown by different studies, while the specificity can be near 100% if conducted under optimal conditions [56,57,58,59,60,61,62,63,64].

Various platforms, from conventional PCR to quantitative real-time PCR (qRT-PCR), nested PCR, multiplex PCR, and loop-mediated isothermal amplification (LAMP) PCR, have been reported to demonstrate variable sensitivity. Still, none of them are free from limitations. Removal of background human DNA from blood specimens [65] and a brief culture of blood samples before the PCR reaction (dubbed blood culture PCR [66]) showed an increased sensitivity by several folds. In summary, PCR-based methods are relatively simpler, faster, and more cost-effective than their culture-based counterparts. However, disease detection sensitivity remains an issue to serving as an optimal assay.

### 2.3. Serological Diagnosis

Serological identification of *S. enterica* serovars relies on Kauffman–White classification. Currently available serological tests cannot reliably diagnose enteric fever (specificity is not 100%) as many of the antigens are shared among different *Salmonella* serovars. The major antigens used to differentiate *S.* Typhi and *S.* Paratyphi are often restricted to Vi, lipopolysaccharides (LPS) O, and flagellar H antigens, yet some of the antigens are shared among different *Salmonella* serovars (Table 2). Therefore, unlike blood-culture-mediated diagnostic methods, positive results from serological diagnostic tests are suggestive of enteric fever (Table 1 and Table 2). However, serological tests are simple and quick, which is highly valuable for managing disease in impoverished endemic areas in a timely manner.

Vi antigen is a linear polymer of α-1,4-2-deoxy-2-N-acetylgalacturonic acid [68]. The genes involved in the expression regulation (*tviA*), synthesis (*tviBCDE*), and transport and localization (*vexABCDE*) of Vi polysaccharide are located in the *viaB* locus as part of *Salmonella* Pathogenicity Island 7 (SPI 7) [69]. The synthesis of Vi polysaccharides is also regulated by a global regulator system *rcsABC* located in the *viaA* locus [70]. Vi polysaccharides are exclusively present in *S.* Typhi and *S.* Paratyphi C (as well as *S.* Dublin and *Citrobacter freundii*) while absent from *S.* Paratyphi A and B and NTS [71]. Vi-antigen-based agglutinations have two major limitations, associated with the recent emergence of Vi-negative strains of *S.* Typhi [72] and the requirement of certain environmental cues for Vi antigens to be expressed by Vi-positive *S.* Typhi strains (e.g., higher osmolality) [73,74].

Somatic O-antigen, a portion of LPS, is present on the outer surface of Gram-negative bacteria. *Salmonella* strains fall into 46 O serogroups that differ in types of sugars, their arrangements, and the linkage within and between repeated O-antigen units, contributing to one of the most variable cell constituents, encoded by highly polymorphic *rfb* genes [75], thus providing the basis for serotyping schemes [76,77]. Vi antigen expressed on the bacterial cell surface can interfere with O-antigen-mediated agglutination, which can be overcome by boiling the bacteria culture for 10 min, a procedure that removes heat-labile Vi but not heat-stable O-antigen [78].

Flagella are present on the cell surface of some bacteria, which facilitate bacterial locomotion. Flagellin protein is the main component of the extracellular flagellar filament that is expressed by one of two genes, H1 (*fliC*) and H2 (*fljB*), one at a time, in *Salmonella*, known as phase variation [79]. The major types of flagellar H-antigens present in typhoidal *Salmonella* are shown in Table 2. *S.* Typhi primarily consists of monophasic H:d antigen; however other variants, H:j or H:z66, have also been reported [80,81].

The most commonly used serological assay in the endemic setting is the Widal test, which measures the agglutination of bacterial O and H antigens with antisera specific for these antigens [82,83]. This test should be performed twice to improve test accuracy: once during the acute phase and the other during the convalescent phase of the infection, which can be approximately 10 days apart. The test result is considered positive if there is a four-fold increase in antibody titers between the two tests [84]. However, due to the unique circumstances posed in endemic areas, a single Widal test is widely used in the field, especially during the early phase of acute infection [85,86]. The interpretation of a single Widal test is complicated by various background antibodies in people of different endemic areas, necessitating determining the cutoff values of antibodies level for determining a positive result [87]. Therefore, when optimum cutoff values tailored for the particular endemic regions are implemented, the specificity and sensitivity of the Widal test can be significantly improved and is better than most of the available rapid diagnostic tests (RDTs) such as Tubex and Typhidot [88]. However, caution should be taken as some other bacteria, as listed above, also express O- and/or H-antigens, which can result in false-positive results [89].

Several RDTs evaluating the presence of enteric-fever-specific immunodominant antigens have been developed to meet the speedy diagnosis requirement in endemic areas [90]. The most commonly used RDTs are Tubex and Typhidot. Tubex detects anti-O9 IgM antibodies in *S.* Typhi [91] and anti-O2 antibodies in *S.* Paratyphi [92]. Typhidot detects IgM and IgG antibodies against the 50-kDa outer membrane protein of *S.* Typhi [93,94]. IgM detection is the most suitable marker for diagnosing acute infection among people who have not previously been infected with *S.* Typhi and *S.* Paratyphi and have not been vaccinated with the Ty21a live attenuated vaccine. IgG detection suggests reinfection in convalescent patients, infection in vaccinated people, or asymptomatic carriers. These tests showed 80–90% specificity and 70–80% sensitivity [90], supporting a possibility of more extensive use of these tests in clinical diagnostic laboratories in endemic regions where a rapid point-of-care (POC)-compatible test is desired for timely management of the disease.

## 3. Future Directions for New Diagnostic Development

Advancing our understanding of host–pathogen interactions, with an emphasis on the bacterial antigens involved, and host responses against the pathogens during various stages of infection will help us discover a diagnostic biomarker(s) suitable for new diagnostic methods, with higher specificity and sensitivity tailored for patient circumstances associated with vaccination and past-infection history.

### 3.1. Overview of Host–Pathogen Interactions

The genome size of *S.* Typhi is approximately 4.8 Mb, producing around 4700 proteins, almost 90% of which are highly shared with NTS, such as *S.* Typhimurium [95,96,97,98]. The *S.* Typhi specific genome consists of 300–400 genes and is characterized by an accumulation of around 200 pseudogenes, also shared in the *S.* Paratyphi genome [95,99,100]. Some of these pseudogenes have functional homologs in NTS serovars that have roles in intestinal colonization. This may partly explain why typhoidal *Salmonella* favors systemic sites in contrast to NTS [101]. Different pathogenic *Salmonella* strains have evolved by acquiring virulence genes located in the loci called *Salmonella* pathogenicity islands (SPIs), prophages, and plasmids [102,103,104]. Various SPIs of varying sizes have been identified, and they encode a cluster of virulence factors that have a role in adhesion, invasion, survival, and toxin production. For example, SPI-1 and SPI-2 encode the type III secretory systems (T3SSs) and many effector proteins. SPI-1 has a role in the invasion of bacteria into intestinal epithelial cells, while SPI-2 is essential for the survival and replication of bacteria within phagocytes. While many SPIs are shared between typhoidal *Salmonella* and NTS, *S.* Typhi has four relatively unique SPIs: SPI-7, 8, 15, and 18 [96]. *S.* Typhi encodes relatively unique virulence factors, such as the virulence (Vi) capsule [71] and a type IVb pilus [105] encoded by SPI-7, the largest SPI, hemolysin (HlyE) encoded by SPI-18 [106], and the typhoid toxin [107,108], among others.

Following ingestion, *Salmonella* can adhere to the mucosa in the small intestine and invade epithelial cells or be taken up by microfold (M) cells [109]. Bacteria enter M cells through receptor-mediated endocytosis and other epithelial cells through an SPI-1-mediated process [110,111]. Postinvasion, bacteria released from intestinal cells are engulfed by phagocytes, primarily by tissue macrophages in the lamina propria. Despite bactericidal activities triggered by host cells, *S.* Typhi can survive and replicate in macrophages by employing SPI-2 virulence factors. Some macrophages then enter the bloodstream through the lymphatic system. This process can result in transient bacteremia, followed by the dissemination of bacteria to the reticuloendothelial system involving the liver, spleen, bone marrow, and gallbladder, all of which can happen within 24 h of pathogen ingestion [40]. During the incubation period, the bacteria markedly replicate in these organs; in some cases, they shed back into the bloodstream, causing secondary bacteremia, which is usually associated with enteric fever symptoms, although bacteria numbers in blood are generally low [112].

Typhoidal *Salmonella* can induce mucosal, humoral, and cell-mediated immune responses in the host [113,114]. The bacteria contain specific pathogen-associated molecular patterns that are recognized by the host innate immunity components, such as Toll-like receptor (TLR) and NOD-like receptor (NLR), on different cell types [115]. For example, flagellin, a protein component of bacterial flagella, is recognized by TLR5 [116], while bacterial DNA can activate TLR9 [117]. The lipid A and lipoprotein moieties of lipopolysaccharide (LPS) stimulate TLR4 and TLR2, respectively [118,119]. Similarly, type IVb pilus has a role in the invasion of human cells [120]. In the case of noninvasive NTS, these interactions lead to the activation of proinflammatory responses localized in the intestine, with the consequence of rapid onset of diarrhea within 12–72 h. However, typhoidal salmonellae typically do not trigger a proinflammatory response [121], where Vi CPS and the typhoid toxin play a role in inhibiting host immune responses by hindering the PAMPs, such as LPS O-antigen, and altering recruited immune cell function and/or depleting those cells, respectively [108,118,122,123,124]. The absence of profound inflammatory responses in infection with typhoidal *Salmonella* is also likely associated with only a transient presence in the circulation while maintaining its intracellular lifecycle in the reticuloendothelial system for most of its infectious cycle [118,125]. In *S.* Paratyphi A, which does not encode the genes for Vi CPS, a very long O-antigen plays a similar role to Vi CPS of *S.* Typhi, which helps evade host innate and adaptive immune responses [126].

### 3.2. Emerging Diagnostic Methods

New biomarker discovery efforts based on proteomics, transcriptomics, and metabolomics have been among the most widely investigated approaches, which is discussed in this section. Using these approaches, investigators have sought biomarkers specific to acute enteric fever patients, allowing them to differentiate these patients from other infectious disease patients and healthy individuals. There are numerous challenges associated with discovering ideal enteric fever biomarkers, which include rather less straightforward validation methods stemming from the lack of a reference standard. Since no single current diagnostic method is perfect, a composite reference standard (CRS) that combines multiple diagnostic tests has been proposed to overcome such limitations [127]. Alternatively, a computational model using Bayesian probability has also been proposed to estimate the accuracy of enteric fever diagnostic tests [128,129,130,131]. Such approaches have revealed a better coverage of actual patients, indicating a promise of these methods based on estimated higher specificity and sensitivity. Furthermore, a lack of an animal model recapitulating the entire infectious life cycle of typhoidal Salmonellae has hampered researchers in understanding the pathogenic mechanism and, thus, exploring novel biomarkers. Still, additional efforts are needed to find an ideal biomarker(s) that is expressed early in the infection stage, indicates drug-resistance profiles, and clearly distinguishes acute infection from subclinical infections or chronic carriers that are prevalent in endemic areas.

#### 3.2.1. Protein Biomarkers

High-throughput approaches, such as conventional and modified proteomics and immunoscreening, have been used to discover immunodominant antigen signatures associated with enteric fever, for instance, protein microarrays, where the whole proteome of *Salmonella* expressed in *E. coli* was probed with enteric fever patient samples to screen for the presence of specific immunodominant bacterial antigen signatures [132,133,134], and immunoaffinity proteomics-based technology (IPT), where columns were packed with enteric fever patient antibodies and probed with bacterial antigens to discover bacterial antigens highly immunogenic in enteric fever patients [135]. Mass spectrometry-based proteomics was followed to identify the bound bacterial proteins.

Studies using liquid chromatography–mass spectrometry (LC–MS) have identified several hundred *Salmonella* antigens; some of them have been further demonstrated using more conventional approaches such as Western blotting for their differential diagnostic potential of acute enteric fever [136]. Some immunogenic bacterial antigens discovered from these studies are outer membrane proteins OmpA and OmpC; virulence factors PagC, CdtB, PltA, and HlyE; chaperone GroEL; locomotor protein flagellin; fimbrial subunits SthA and SthD; LPS, among others. Some of these potential biomarkers include typhoidal *Salmonella* proteins such as CdtB, PltA, and HlyE. Intracellular pathogens like *S.* Typhi and *S.* Paratyphi dynamically change their gene expressions during infection, which are drastically different from the ones expressed during in vitro laboratory culture conditions (e.g., LB). Consistently, many *S.* Typhi genes are known to be exclusively expressed from intracellularly located bacteria (e.g., CdtB, PltA, PltB, HlyE, and SPI effector proteins) [107,137,138]. To better reflect bacterial antigens expressed during human infection, in-vivo-induced antigen technology (IVIAT) has also been performed to screen a library of *Salmonella* proteins to identify bacterial antigens expressed during human infection [139]. Improving the limit of detection remains to be resolved for obtaining a more comprehensive dataset.

Immunoglobulin isotypes against bacterial antigens found in infected people can be used as an indicator to reflect various infection stages. For example, the presence of IgM and IgA against *S.* Typhi antigens without high levels of IgG suggests acute infection [135]. In contrast, higher levels of IgG against bacterial antigens can indicate acute and chronic infection stages, depending on the vaccination and preinfection history of suspected patients. *Salmonella*-activated lymphocytes secreting mucosa-derived IgA were detected in peripheral blood as early as 3 days after infection, reaching peak level by Day 7 [140,141]. In some cases, modifications in procedures, such as the isolation and transient in vitro culture of activated lymphocytes, have been implemented to maximize IgA detection. Similarly, antibodies were detected from lymphocyte supernatant (antibodies in lymphocyte supernatant or ALS) [142]. Such methods improved the limit of antibody detection compared to the procedures using patient plasma samples, indicating a promise of early disease diagnosis.

Antigens such as HlyE and LPS exhibit high diagnostic potential [143]. HlyE is relatively unique to *S.* Typhi and *S.* Paratyphi since this gene product is absent from most NTS, including *S.* Typhimurium and *S.* Enteritidis [106]. Although HlyE homologs are present in *E. coli*, IgA-HlyE could discriminate enteric fever from other febrile infections, including NTS [144]. Various studies have demonstrated that elevated levels of both IgA and IgG in the tested patient samples, which show a promise in differentiating acute enteric fever patients who may be reinfected or vaccinated [144,145,146,147,148]. Furthermore, a noninvasive method using saliva samples, measuring IgA-HlyE antibodies via an ELISA-based method [149], was also tested.

Additionally, multiple antigens have been investigated simultaneously to achieve the highest accuracy in bacterial detection. One such assay format included a multiplex immunochromatographic strip detecting both IgA-HlyE and IgA-LPS, which exhibited a high diagnostic accuracy [145,150]. Here, HlyE can discriminate enteric fever from other febrile infections, while LPS distinguishes enteric fever from healthy groups. Similarly, several studies have demonstrated the promise of CdtB, an enzymatic subunit of typhoid toxin that is produced only during infection, as a biomarker for acute enteric fever diagnosis. Significantly increased levels of IgG-CdtB in enteric fever patient plasma and ALS samples were detected, indicating its serodiagnostic potential [132,133,135]. IgM-CdtB has also been detected but at a lower level using standard ELISA methods [151]. While typhoid toxin orthologs are encoded in the genomes of some NTS serovars, their target host cells are intestinal epithelial cells that produce different clinical presentations [123]. Amino acid sequence variations on glycan receptor binding pockets of typhoid toxin orthologs are responsible for a narrow host cell tropism [123]. To make this biomarker a more reliable diagnostic method, some changes should be made to improve the detection limit and/or use it as part of signatures that can be combined with other biomarkers, such as Vi polysaccharides [152], LPS, and other membrane components [142].

ELISA and an immunodot blot method called TPTest, evaluating IgA titers against membrane components of *S.* Typhi and *S.* Paratyphi using ALS samples, exhibited 78–97% specificity and 100% sensitivity in detecting the bacteria [129,142,153]. TPTest is advantageous in differentiating acute infection from convalescence, which would make this test a valuable tool for disease diagnosis in endemic areas [153]. With additional improvements, these biomarkers could be developed as POC-compatible rapid diagnostic methods.

#### 3.2.2. Nucleic Acid Biomarkers

Using transcriptional profiling approaches such as microarray hybridization and RNA-Seq, gene expression profiles of bacterial and host cells that occurred during various infection stages have been analyzed, resulting in the discovery of new biomarkers. For instance, microarray analysis detected 2026 *S.* Typhi genes (~44% of the genome) from infected blood cells, with 141 transcripts upregulated, including PhoPQ regulatory genes, the typhoid toxin, and HlyE [154]. Microarray analysis has also been exploited to identify host genes. Relative gene expression pattern analysis of peripheral blood samples reflecting acute, recovery, convalescent, and uninfected groups for enteric fever produced reproducible blood signatures specific to the disease [155]. The transcripts identified in this study were correlated to clinical parameters [155]. Investigators of this study noted the need for careful data interpretation to avoid a possible cumulative effect of responses as blood represents both a pool and a migration compartment for various immune cell types.

A more recent study investigated five host genes as a signature (STAT1, SLAMF8, PSME2, WARS, and ALDH1A1) that was able to identify enteric fever with 88% specificity and 97% sensitivity [156]. If these signatures are also observed in different endemic areas, amplification of such genes by qPCR-based diagnostic assay can be developed as a promising diagnostic method. A novel method named miniature NMR (μNMR), detecting bacterial mRNA using magneto-DNA probes, has been proposed; it is capable of detecting up to 1 CFU/mL *S.* Typhi and *S.* Paratyphi [140], indicating the promise of developing an ultrasensitive detection method. Some tweaks may be required as detecting mRNA may be more challenging than detecting DNA due to the relatively unstable nature of bacterial mRNA.

#### 3.2.3. Metabolite Biomarkers

To understand specific metabolic changes occurring in enteric fever patients, metabolomics has been conducted. For instance, comparative analysis of metabolites between patient plasma samples infected with *S.* Typhi or *S.* Paratyphi and control samples via gas chromatography–mass spectrometry approaches identified 695 distinct metabolite peaks [157]. A combined analysis of highly reproducible top 6 peaks, reflecting ethanolamine, gluconic acid, monosaccharide, phenylalanine, pipecolic acid, and saccharide, was able to distinguish typhoid fever patients from paratyphoid fever patients and healthy individuals. A tight correlation of other metabolites, such as iron and tryptophan, to typhoid fever has also been demonstrated. *S.* Typhi induced a rapid decline of plasma iron levels and the retention of iron inside tissue macrophages through the upregulation of hepcidin [158]. Acute infection with *S.* Typhi generates a specific interferon signature that alters tryptophan catabolic pathways, leading to the pathogenesis of typhoid fever [159]. Identifying any single or combined differentially induced metabolites during the different infection stages could discover promising novel biomarkers. Further studies covering sufficient sample size and other febrile disease samples also prevalent in endemic areas are anticipated to result in a much-improved enteric fever diagnostic method.

## 4. Concluding Remarks

Current enteric fever diagnostics such as culture-based methods exhibit superior specificity but suffer from low sensitivity and relatively slow turnaround time. PCR-based nucleic acid detection methods are reasonably rapid but require trained personnel and special equipment to conduct. Serological methods exemplified by the Widal test are quick and, therefore, highly POC-compatible in endemic areas but exhibit modest specificity and sensitivity. Toward the establishment of optimal diagnostic methods, several high-throughput approaches have been carried out to search for bacterial and host biomarkers that are relatively unique for enteric fever and differentiating acute, recovery, and convalescent infection stages. The currently available data are not sufficient to point out a single ideal biomarker. However, these results will serve as the groundwork for future efforts. For instance, if we decide to go with a single or few biomarkers available from the completed approaches, a breakthrough from standard detection methods leading to ultrasensitive detections of such biomarkers should be made. If we decide to use traditional detection methods, a cost-effective signature biomarker panel that differentiates enteric fever from other febrile diseases and healthy/healthy-recovered individuals who have been previously exposed to pathogens should be established. Moreover, the discussed high-end technologies (e.g., proteomics, transcriptomics, and metabolomics) are not compatible with the lack of infrastructure in endemic areas. Therefore, there is a need to identify cheaper and simpler methods or convert these technologies into something more accessible. The reality is that the most important factor in diagnosing *S.* Typhi and *S.* Paratyphi infections in endemic regions is finding something that is inexpensive and easy to use. Lastly, although it is less straightforward, future efforts should include developing diagnostic methods detecting healthy/asymptomatic chronic carriers of these human-specific pathogens.

## Figures and Tables

**Table 1:** Diagnostic tests for acute enteric-fever-suspected patients (fever ≥38 °C for ≥3 days).

Methods	Advantages	Limitations	Adjustments *
Blood/bone marrow culture (confirmed by positive culture results)	a. 100% specificity. b. Isolated bacteria can also be used for subsequent antibiotic susceptibility tests and molecular characterization.	a. Low sensitivity: ~50% blood culture, ~80% bone marrow culture. b. Bone marrow collection is invasive. c. Time-consuming (≥48 h). d. These test methods require trained personnel and infrastructure, which are not necessarily common in endemic areas.	a. Use of larger sample volume. b. Lowering bactericidal activity of blood (by supplementing bile salt or sodium polyethanol sulfonate, removing serum, or diluting blood). c. Lysis of blood cells to release bacteria.
Bile/stool culture (suggested by positive results)	a. Isolated bacteria can also be used for subsequent antibiotic susceptibility tests and molecular characterization.	a. Bile/stool positive can also be due to chronic infection. b. Test methods show moderate sensitivity and specificity. c. Time-consuming (≥48 h). d. These test methods require trained personnel and infrastructure, which are not necessarily common in endemic areas.	a. Use of larger sample volume.
Bacterial nucleic acid detection (suggested by positive results)	a. Nucleic acid tests can also detect non-culturable/dead bacteria (beneficial for patients who already take antibiotics before office visits).	a. Moderate sensitivity and specificity.	a. Bacterial nucleic acids can be enriched by removing human DNA and transient culture.
Serological tests (suggested by positive results)	a. Quick turnaround time associated with high point-of-care compatibility. b. Some serological tests are simple, quick, and inexpensive.	a. Cross-reactivity. b. Moderate sensitivity and specificity.	a. Use of isolated or cultured bacteria.

* Test success rates can be improved by adjustments.

**Table 2:** Serological identification of *Salmonella*.

Serovar Name	LPS O Ag	Flagella H Ag	Vi Ag *	Cross-Reactivity
*S.* Typhi	9	d	Positive	O9 Ag is present in *S.* Enteritidis, *S.* Dublin, and *S.* Gallinarum. Vi Ag is present in *S.* Paratyphi C, *S.* Dublin, and *Citrobacter freundii*.
*S.* Paratyphi A	2	a	Negative	
*S.* Paratyphi B	4	b	Negative	O4 Ag is present in *S.* Typhimurium.
*S.* Paratyphi C	6/7	c	Positive	O6/7 Ags are present in *S.* Choleraesuis. Vi Ag is present in *S.* Dublin, *Citrobacter freundii*, and *S.* Typhi.

* Vi antigen is mainly used to screen for chronic carriers [67].

## Data Availability

This paper contains all data.
